# Effectiveness and cost-effectiveness of outpatient physiotherapy after knee replacement for osteoarthritis: study protocol for a randomised controlled trial

**DOI:** 10.1186/s13063-016-1418-x

**Published:** 2016-06-13

**Authors:** Vikki Wylde, Neil Artz, Elsa Marques, Erik Lenguerrand, Samantha Dixon, Andrew D. Beswick, Amanda Burston, James Murray, Tarique Parwez, Ashley W. Blom, Rachael Gooberman-Hill

**Affiliations:** Musculoskeletal Research Unit, School of Clinical Sciences, University of Bristol, Learning and Research Building (Level 1), Southmead Hospital, Bristol, BS10 5NB UK; School of Health Professions, University of Plymouth, Peninsula Allied Health Centre, Derriford Road, Plymouth, Devon PL6 8BH UK; National Institute for Health Research (NIHR) Collaboration for Leadership in Applied Health Research and Care West (CLAHRC West), University Hospitals Bristol NHS Foundation Trust, Upper Maudlin Street, Bristol, BS2 8HW UK; North Bristol NHS Trust, Brunel Building, Southmead Hospital, Bristol, BS10 5NB UK; Emersons Green Independent Treatment Centre, The Brooms, Emersons Green, Bristol, BS16 7FH UK

**Keywords:** Total knee replacement, Physiotherapy, Function, Randomised controlled trial, Economic evaluation

## Abstract

**Background:**

Primary total knee replacement is a common operation that is performed to provide pain relief and restore functional ability. Inpatient physiotherapy is routinely provided after surgery to enhance recovery prior to hospital discharge. However, international variation exists in the provision of outpatient physiotherapy after hospital discharge. While evidence indicates that outpatient physiotherapy can improve short-term function, the longer term benefits are unknown. The aim of this randomised controlled trial is to evaluate the long-term clinical effectiveness and cost-effectiveness of a 6-week group-based outpatient physiotherapy intervention following knee replacement.

**Methods/design:**

Two hundred and fifty-six patients waiting for knee replacement because of osteoarthritis will be recruited from two orthopaedic centres. Participants randomised to the usual-care group (*n* = 128) will be given a booklet about exercise and referred for physiotherapy if deemed appropriate by the clinical care team. The intervention group (*n* = 128) will receive the same usual care and additionally be invited to attend a group-based outpatient physiotherapy class starting 6 weeks after surgery. The 1-hour class will be run on a weekly basis over 6 weeks and will involve task-orientated and individualised exercises.

The primary outcome will be the Lower Extremity Functional Scale at 12 months post-operative. Secondary outcomes include: quality of life, knee pain and function, depression, anxiety and satisfaction. Data collection will be by questionnaire prior to surgery and 3, 6 and 12 months after surgery and will include a resource-use questionnaire to enable a trial-based economic evaluation. Trial participation and satisfaction with the classes will be evaluated through structured telephone interviews. The primary statistical and economic analyses will be conducted on an intention-to-treat basis with and without imputation of missing data. The primary economic result will estimate the incremental cost per quality-adjusted life year gained from this intervention from a National Health Services (NHS) and personal social services perspective.

**Discussion:**

This research aims to benefit patients and the NHS by providing evidence on the long-term effectiveness and cost-effectiveness of outpatient physiotherapy after knee replacement. If the intervention is found to be effective and cost-effective, implementation into clinical practice could lead to improvement in patients’ outcomes and improved health care resource efficiency.

**Trial registration:**

ISRCTN32087234, registered on 11 February 2015.

## Background

Knee osteoarthritis (OA) is the leading cause of pain and disability in older people [1]. If pharmacological and conservative treatments do not relieve symptoms, primary total knee replacement (TKR) is commonly performed. In 2013, over 70,000 TKR operations were performed in the National Health Service (NHS), with 96 % of procedures subsequent to OA [2]. Although the operation is effective for many patients, a considerable proportion of patients experience long-term pain and functional limitations after surgery [3, 4]. An estimated 20 % of patients report long-term pain after TKR [3] and 52 % of patients report functional limitations, compared to 22 % of age- and gender-matched people without TKR and no previous history of knee disorders [4]. Evidence also suggests that many patients do not return to more demanding activities after TKR, such as gardening [4, 5], kneeling [6], sports [7] and valued leisure activities [8].

Prior to consideration of surgical intervention, exercise can be beneficial in improving knee function for patients with OA [9], and exercise is recommended as a conservative strategy to manage the symptoms of OA [10]. Once listed for TKR, a pre-operative exercise programme may be offered to patients with the aim of maximising post-operative recovery and function. However, systematic reviews have found that post-operative function is not improved by pre-operative exercise [11–13]. This indicates the need to evaluate rehabilitation interventions delivered after surgery. Physiotherapy in the immediate post-operative period is a standard component of post-surgical care. However, rehabilitation at this early recovery stage is predominately targeted at joint mobilisation and the achievement of short-term functional goals relating to hospital discharge. Therefore, the optimal time to deliver interventions targeted at improving long-term function after TKR may be in an outpatient setting after hospital discharge. However, research has identified international variation in the provision of these services [14–18], and no UK guidelines for outpatient physiotherapy after TKR currently exist.

In a systematic review and meta-analysis of six randomised trials published prior to 2007, outpatient physiotherapy was found to improve physical function in the first 3–4 months after TKR [19]. An recent updated systematic review and meta-analysis including 17 randomised trials also found evidence of short-term functional benefits [20]. However, both reviews found there was insufficient evidence to determine whether benefits were retained in the longer term. Physiotherapy should address patient expectations [21], and the key expectations of patients undergoing TKR surgery relate to long-term functional and pain outcomes [22, 23]. Therefore, there is a need to evaluate the individual and societal burden of long-term pain and functional limitations after TKR. Functional outcomes after TKR plateau at around 12 months post-operative [24] and consequently trials with at least 12 months’ follow-up are needed to establish the long-term effectiveness of post-discharge physiotherapy after TKR.

The aims of this randomised controlled trial (RCT) are to determine the clinical effectiveness and cost-effectiveness of a novel group-based outpatient physiotherapy intervention consisting of task-oriented and individualised exercises for improving long-term function for NHS patients receiving primary TKR because of OA.

## Methods/design

The ARENA study (Activity-orientated REhabilitation following kNee Arthroplasty) is a multi-centre, pragmatic, superiority RCT to evaluate the long-term effectiveness and cost-effectiveness of outpatient group-based physiotherapy after TKR in addition to usual care. The trial has been approved by the National Research Ethics Committee South West – Central Bristol (reference 14/SW/1144) and is registered on the ISRCTN registry (ISRCTN32087234). The trial is informed by a feasibility study of the RCT with 46 patients and a 6-month post-operative follow-up [25]. In this study, the intervention was developed and the feasibility of delivering the intervention to patients with TKR in the context of a RCT was evaluated. The trial was developed in collaboration with the Patient Experience Partnership in Research (PEP-R) group [26], a dedicated, specialised patient-involvement group comprising 16 patients with musculoskeletal conditions, many of whom have had joint replacement.

### Study duration

Recruitment of the trial began in March 2015 and 12-month follow-up for all participants is anticipated to be complete by December 2017.

### Participant recruitment

NHS patients will be screened and recruited from pre-operative assessment clinics at two elective orthopaedic centres in Bristol: Southmead Hospital and Emersons Green Independent Treatment Centre. Inclusion criteria include NHS patients aged 18 years or older who are listed for primary TKR due to OA. Exclusion criteria include (1) patients listed for TKR for reasons other than OA, (2) patients listed for revision TKR, (3) inability to participate in exercise for medical reasons such as unstable cardiovascular or severe neurological conditions, (4) unable or unwilling to attend physiotherapy classes after surgery, (5) unable or unwilling to provide informed consent, (6) inability to understand English because not all the questionnaires have been translated and validated into other languages, and (7) post-operative complication(s) or interventions within the first 2 weeks of surgery which would preclude participation in the physiotherapy classes; for example, prosthetic joint infection or manipulation under anaesthetic. Participation in pre-operative physiotherapy was not a selection criterion for this trial. In order to explore whether the patients who are enrolled in the trial are representative of those undergoing TKR, anonymised data about age and gender will be recorded for all eligible patients. After patients who wish to participate in the trial have provided written, informed consent, they will be asked to complete a pre-operative questionnaire.

### Randomisation

Participants will be randomised with a 1:1 treatment allocation to the intervention group or usual care group 2 weeks after TKR. Prior to randomisation, eligibility criteria will be reassessed through contact with participants and review of operation notes. Randomisation with allocation concealment will be conducted by means of a computer-generated code, administered centrally and communicated via the Internet (through the Bristol Clinical Trials and Evaluation Unit). Randomisation will be stratified by pre-operative function measured by the Lower Extremity Functional Scale [27] (categorised as high or low function based on mean scores from a published study with a similar patient cohort [28]) and recruitment centre (Southmead Hospital or Emersons Green Treatment Centre). Blinding of participants and trial personnel to treatment allocation will not be possible due to the nature of the intervention.

### Usual care

At both recruitment centres, usual physiotherapy after discharge following TKR surgery comprises information and advice on knee-specific and functional exercise. Referral for outpatient physiotherapy is on a needs-only basis when deemed appropriate by a member of the clinical care team. Prior to hospital discharge, patients are assessed on an individual basis by the inpatient physiotherapy team at each centre, and patients with poor range of motion or muscular weakness are referred for outpatient community-based physiotherapy. This referral is at the discretion of the hospital’s physiotherapy or orthopaedic team. General practitioners (GPs) and consultants can also refer patients for outpatient physiotherapy as appropriate. Use of physiotherapy services by participants in both trial arms will be recorded in the trial questionnaires.

### Intervention

Participants allocated to the intervention group will receive the intervention in addition to usual care. The intervention is a novel weekly 1-hour physiotherapy class, starting 6 weeks after surgery and then on a weekly basis over 6 consecutive weeks. Previous trials have evaluated outpatient physiotherapy after TKR [20]; however, this intervention is novel as it embeds individualised exercises within a group-based task-oriented exercise circuit. The design of the intervention was informed by intervention development work, which included a systematic review [20], survey of current practice [14] and a feasibility study to pilot the intervention [25]. The intervention consists of task-orientated exercises and individualised exercises. Previous research has demonstrated the benefits of task-orientated, functional exercises [19] and the importance of improving patients’ ability to participate in ‘valued activities’ [8, 29]. Task-orientated exercises have been found to be more effective than traditional exercises, such as range of motion exercises, in improving function and participation after TKR [19] and other conditions such as stroke [30]. The inclusion of individualised exercises aims to address patients’ expectations, empower people to take an active role in rehabilitation, and increase self-efficacy [31, 32]. Delivery is in a group-based setting, which can be a cost-effective way to deliver rehabilitation without compromising effectiveness [33–35]. There is currently no consensus on the optimal treatment frequency or number of sessions for physiotherapy after TKR [36]. The intensity of our intervention was informed by previous research [20], combined with the need to develop an intervention that would be deliverable within the NHS if proven to be effective.

Classes will be held at set weekly times in an outpatient gymnasium at a large NHS hospital and up to a maximum of 12 patients can attend a class. Classes will be supervised by a physiotherapist and physiotherapy technician/assistant, and will run on a rolling system so that new patients can join the classes each week as other patients finish the class. Participants will be reimbursed travel costs that are incurred for their travel to attend classes. Before attending the physiotherapy classes, participants will be asked to complete an Activity Goal Form which involves identifying two functional goals that they would like to achieve. In their first class, each participant will have a discussion with the physiotherapist about these goals to develop two individualised exercises which they begin in their second class. Participants will also be given an exercise booklet in which they record details about their weekly progress in the class.

Each class will involve a short warm up, after which patients follow a simple exercise circuit. The 5-min warm up involves participants mobilising around the gym circuit at a steady pace. This is interspersed with gentle upper limb and lower limb movements including shoulder circumduction, elbow flexion and extension, hip and knee flexion, and ankle circumduction exercises, as each participant is able. The exercise circuit involves 12 exercise stations, including two stations dedicated to individualised exercises. An overview of the exercises stations is provided in Table 1. Four minutes is allocated to each station to provide patients with sufficient time to carry out the exercises at their own pace. The exercises are designed to increase lower limb strength, balance, function and confidence using specific and task-related activities. A selection of graded exercises is provided at each station to enable the patients to exercise at a level suitable to their ability. During weeks 2–6, the exercises are progressed on an individual basis through discussion with the physiotherapists.

**Table 1:** Brief description of exercises at each station within the physiotherapy class

Station and exercise	Description	Task
1. Bed-based exercises	Low-grade exercises including knee flexion and extension range of motion (2 × 8 repetitions), quadriceps strengthening (2 × 8 repetitions), hamstring strengthening (2 × 8 repetitions), quadriceps stretching (2 × 5 repetitions), and hamstring stretching (2 × 5 repetitions). Progressions include increasing number of repetitions, changing position, and addition of resistance bands or ankle weights	Maintain/improve knee range of motion and strength, simulation of kicking/swimming
2. Getting in/out of bed	Practice turning from back to side and to sitting. Then stand from sitting. Return to sitting then lying (2 × 5 repetitions). Progression includes bridging then sit to stand	Practice transferring in and out of bed log rolling, and sit to stand
3. Balance tasks	Balanced-based exercises including single leg stance (3 × 30 sec) and wobble board (3 × 30 sec). Progressions including increasing duration and including upper limb actions such as throwing, catching and reaching	Improve static and dynamic balance. Falls prevention
4. Stair exercises	Stepping up and down on stairs of varying height (3 × 8 step-ups). Progression includes using higher step	Stair ascent and descent
5. Individualised exercise 1	Exercise designed specifically for individual patients	Individual task
6. Walking exercises	Walking practice (gait re-education). Progressions including from aided to unaided, side stepping, walking over uneven surfaces, walking carrying objects	Walking. Falls prevention
7. Squatting and crouching	Mini and semi-squats (3 × 8 repetitions). Squats can be performed with the assistance of chairs and gym ball. Progressions including increasing the depth of squat and crouching	Squatting and crouching down
8. Cycling	Static bike (1 min cycling followed by 30 sec rest and then repeat). Progressions include increasing resistance and duration of cycling	Improve cardiovascular fitness and knee range of motion
9. Gardening/kneeling	Replicating digging action using stepper (3 × 8 repetitions) with progression including increasing resistance of stepper. Kneeling onto cushioned or hard surfaces (3 × 8 repetitions) with progression including full kneeling and high kneeling. Activities to desensitise the knee joint such as light pressure using different textures (30–60 sec)	Improve kneeling ability
10. Lunges	Mini lunges (2 × 8 repetitions). Progressions include depth of lunge, lunge walking and lunge to bowling or picking up objects	Improve knee strength and ability to picking objects up from floor
11. Individualised exercise 2	Exercise designed specifically for individual patients	Individual task
12. Treadmill/cross-trainer	Practice straight-line walking. Progressions including increase in speed and incline of treadmill, use of cross-trainer	Walking on flat and uphill. Jogging
		Improve cardiovascular fitness

Towards the end of the intervention, the physiotherapist will spend time with each participant to develop their home exercise programme. Every participant will be provided with an individualised written plan detailing the key exercises to continue at home. Advice on number of repetitions and how often the exercises should be performed will be provided, tailored to the participant’s individual ability.

### Assessment times

Participants will be followed up for 12 months after TKR. Participants will be asked to complete questionnaires before their surgery and at 3 months, 6 months and 12 months after their surgery. Post-operative questionnaires will be posted to participants, followed by a postal reminder and a telephone call to non-responders.

**Fig. 1:**
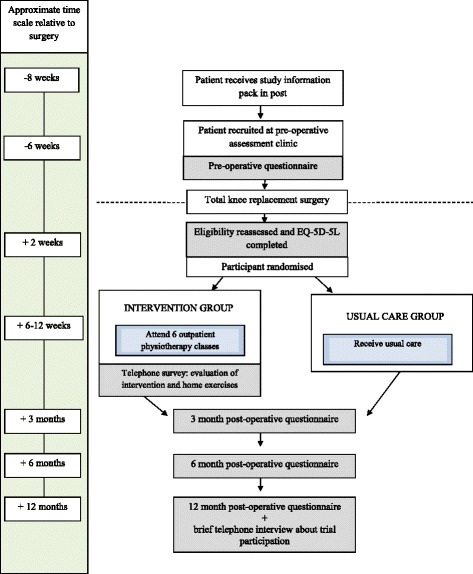
Flow chart of trial participation

In addition to the questionnaires, further information will be collected by telephone. At 2 weeks post-operative, all participants will be telephoned and asked to complete the five-level EuroQol questionnaire (EQ-5D-5 L) [37]. Patients who attend the intervention will be telephoned 1 month after completion of the classes to complete a short telephone survey to evaluate the classes and home exercise programme. All participants who complete the 12-month follow-up of the trial will be contacted by telephone and asked if they would be willing to complete a telephone survey about the experience of trial participation. A flow chart of trial participation is provided in Fig. 1.

### Primary outcome

The primary outcome is the Lower Extremity Functional Scale (LEFS) [26] at 12 months post-operative. The LEFS is a validated 20-item questionnaire which assesses lower limb function and difficulty in performing everyday tasks. The LEFS has been recommended as the outcome measure of choice to measure function in patients with knee OA and those undergoing joint replacement because of its good psychometric properties and minimal floor and ceiling effects [38–40].

### Secondary outcomes

Secondary outcomes include quality of life, knee pain, knee function, depression, anxiety and patient satisfaction. These outcomes will be assessed using the following validated measures at 3, 6 and 12 months post-operative, except where indicated:

- EQ-5D-5L [37]: measures quality of life and allows derivation of incremental quality adjusted-life years (QALYs) for the economic evaluation. In addition to the standard assessment time points, the EQ-5D-5L will be administered by telephone at 2 weeks after surgery
- LEFS at 3 months and 6 months post-operative
- Knee Injury and Osteoarthritis Outcome Score (KOOS) [41]: assesses pain, other symptoms, function in daily living, function in sport and recreation and knee-related quality of life
- Hospital Anxiety and Depression Scale (HADS) [42]: assesses symptoms of depression and anxiety
- Self-Administered Patient Satisfaction Scale for Primary Hip and Knee Arthroplasty [43]: assesses satisfaction with the outcome of knee replacement
- Likert-type scale for satisfaction with physiotherapy treatment received

### Resource use

Resource-use data will be collected from hospital discharge to 12 months post-operative. Resources required to deliver the intervention will be recorded in Case Report Forms by the physiotherapists delivering the intervention. Patient self-report use of services will be collected through patient-completed resource-use questionnaires. The questions will cover the use of additional physiotherapy treatments in hospital and community and other therapies prescribed, readmissions, outpatient visits, GP and nurse visits, medication use and use of social services. To allow for a secondary societal analysis, questionnaires will further collect time off work, leisure activities, private expenditures (e.g. privately paid therapies, travel costs to sessions) and informal care. All participants will be provided with a resource-use log to prospectively fill in resources used to aid them in the completion of the resource-use questionnaires [44].

### Patient characteristics

Data on socioeconomic status (marital status, living arrangements, ethnicity, educational attainment, working status), medical co-morbidities (Functional Co-morbidity Index [45]), knee symptoms (LEFS, KOOS), quality of life (EQ-5D-5L), and depression and anxiety (HADS) will be collected in the pre-operative questionnaire to allow adjustment for these variables in the analysis. Data on age and gender will be collected by the researcher at recruitment.

### Patient satisfaction, adherence and perceived value of the physiotherapy classes

Patients who are randomised to the intervention group and attend the physiotherapy classes will be telephoned by a member of the research team 1 month after completion of the classes and asked if they would be willing to answer questions about the physiotherapy classes and home exercise programme. Participants will be asked questions about satisfaction with the classes, ability to achieve personal goals and aspects of the class that they found helpful or unhelpful. To evaluate the home exercise programme, participants will be asked about how often they have been performing their home exercises, any barriers or facilitators to performing the exercises, and any perceived value/benefits of performing the exercises. Responses to the questions will be recorded by a researcher on a standardised proforma.

### Trial evaluation

Brief structured telephone interviews to evaluate trial participation will be conducted with participants after completion of the 12-month post-operative questionnaire. Interview questions will be open-ended and will cover reasons for participating in the trial, experiences of trial participation, and any perceived benefits or negative aspects to participating in the trial. Participants’ responses will be recorded by a member of the research team on a standardised proforma. In addition, patients who self-withdraw from the trial will be asked if they would be willing to discuss their reasons for withdrawal with a member of the research team.

### Adverse events

Data on adverse events and serious adverse events will be collected and closely monitored to ensure the ongoing safety of participants. Adverse events will be recorded on a standardised Adverse Events Report Form. All serious adverse events will be notified to the study sponsor and reviewed by the Trial Steering Committee.

### Sample size

The minimal clinically important difference (MCID) for the LEFS is 9 scale points [26]. In our feasibility study, we observed a pooled standard deviation (SD) of 18.4 and a rate of missing LEFS score of 9 % in the intervention group and 35 % in the usual-care group at 6 months post-operative. For the purposes of the sample size calculation we assumed a similar SD for the LEFS at 12 months post-operative, due to the lack of relevant published data. To account for the uncertainty induced by estimating parameters from a small feasibility study, we adjusted the assessed sample by an inflation factor of 1.12^2^, a value derived from the 80 % upper confidence limit of the SD estimate [46]. We also accounted for a missing data rate of 35 %, although we are implementing additional measures to improve LEFS completion rates (e.g. completion of the primary outcome measure over the telephone with non-responders). Therefore, a sample of 256 patients will allow us to detect a MCID (9 points) in the LEFS between trial arms at 12 months post-operative, assuming a power of 80 %, a two-sided 5 % significance level and accounting for up to 35 % missing data and an ‘inflation factor’ of 1.12^2^.

### Statistical analysis

Analysis will follow a Statistical and Health Economics Analysis Plan which will have been approved a priori by the Trial Steering Committee. Data presentation and analysis will be in accordance with Consolidated Standards of Reporting Trials (CONSORT) guidelines. Baseline characteristics will be reported by trial arm using percentages, means (SDs) or medians (interquartile ranges) as appropriate. The repeated measures of primary and secondary outcomes will be plotted by trial arm.

The main analysis will consist of a linear mixed regression (with random intercept for patient to control for the repeated follow-ups) with an interaction between the intervention effect and the assessment time adjusted for pre-operative function and recruitment centre. The use of these interactions terms will allow us to assess and single out the specific effect of the intervention on LEFS at 12 months post-operative, and then identify the intervention-specific effects at 3 and 6 months post-operative (secondary outcomes). The regression will then be adjusted for imbalanced individual characteristics between arms at baseline. Other types of generalised linear mixed models will be considered if the former approach is not fitting the distribution of the residuals of the regressed (transformed) primary or secondary outcomes. The model will finally be adjusted for type of additional physiotherapy treatment received (physiotherapy received that is not delivered as part of the intervention). The trial is not powered for such adjustments and it will only inform us on their potential impact on the intervention effect.

The analyses will be conducted on an intention-to-treat basis with and without imputation of missing primary outcome data. A multiple imputation (MI) by chained equations under a missing at random framework stratified by randomization arm will be used [47]. These imputed results will be contrasted with other imputation strategies as discussed by White and colleagues [48]. The same modelling strategy will be used to investigate the intervention effect on the KOOS, HADS and patient satisfaction. A sensitivity analysis will investigate the intervention effect using a per-protocol analysis. Information clustering at the recruitment centre level will be addressed using a fixed-effect indicator and clustering of outcomes by surgeon will be investigated and modelled if required. We will also compare the mean/median primary outcome by physiotherapist for patients receiving the intervention and any significant differences will be used to conduct exploratory sub-group analyses. Given the differences in outcomes after TKR for men and women [49], exploratory analysis will be undertaken to investigate the impact of gender.

### Health economic analysis

The primary economic evaluation will follow NICE guidelines [50] and will be a cost-utility analysis (CUA), comparing the incremental costs with incremental QALYs gained, from an NHS and Personal Social Services (PSS) perspective, at 12 months post-operative. Secondary analyses will include taking a societal perspective on costs, and a cost-effectiveness analysis comparing the incremental costs with the primary clinical outcome at 12 months. All analyses will follow a Statistical and Health Economics Analysis Plan.

Resources will be valued using unit cost estimates from national tariffs [51, 52], and liaising with hospital finance departments if necessary. We will assign UK preference-based utility weights [53] to patients’ answers to the EQ-5D-5L questionnaire, and produce unadjusted 1-year QALY scores per arm, using the area under the curve approach [54]. Costs and QALY estimates will then be adjusted for stratification variables, and also for baseline utility [55] for QALYs, using regression methods. Costs and outcomes will be compared between arms to determine dominance, i.e. whether one arm is more effective and costs less than the other. Missing data will be imputed using multiple chained equations [47].

The economic result will be bootstrapped incremental net monetary benefit (INMB) statistics, using a range of societal willingness-to-pay thresholds for a QALY gained. If no arm is dominant, we will compute incremental cost-effectiveness ratios (ICERs) in relation to both outcomes. The primary results will report estimates based on complete datasets with imputed data. We will plot adjusted bootstrapped costs and effects in cost-effectiveness planes and create cost-effectiveness acceptability curves to consider uncertainty around the adoption decision. In sensitivity analysis we will explore sources of methodological and parameter uncertainty, such as costing assumptions.

## Discussion

This project is a fully powered RCT to evaluate the long-term clinical effectiveness and cost-effectiveness of a novel group-based outpatient physiotherapy intervention following TKR in addition to usual care. The trial includes patients with all levels of functional aspirations, and emphasises the concept of participation in valued activities as a basis for effective and appropriate rehabilitation goals. Our approach aims to both improve function for those patients who may otherwise have unfavourable outcomes, and assist patients who are recovering well to achieve more demanding goals.

A strength of this trial is that the design of the intervention and trial were informed by prior feasibility work [25]. In particular, the feasibility work highlighted the importance of complementing the collection of outcome measures by postal questionnaire with telephone calls to maximise completion rates. This has previously been shown to be an effective strategy with a similar patient population [56], although with the increasing uptake of technology, online questionnaires may be a useful alternative data collection method for future trials. Another strength of this trial is the 12-month follow-up period, which will allow assessment of the long-term impact of physiotherapy on patient outcomes and resource use. However, in addition to patient-reported outcome measures, the trial may have benefitted from the inclusion of more objective measures of function, such as muscle strength or gait analysis, as these capture different dimensions of function [57]. Another limitation of the trial which warrants acknowledgement is the lack of blinding, because of the group-based nature of the physiotherapy intervention. Blinding in trials of physiotherapy is often challenging and only a quarter of physiotherapy trials are blinded [58]. There is a risk that lack of blinding will introduce bias as outcome data will be self-reported by participants who will know which treatment they have been allocated to. A recent meta-epidemiology study found that lack of blinding in physiotherapy trials can lead to an underestimation of the treatment effects compared to trials with blinding, although this was not statistically significant [58]. In addition, while the trial is powered for our primary analysis, other analyses are purely exploratory in nature and will be interpreted as such.

The findings of this trial will provide evidence that has the potential to inform physiotherapy service provision for patients after TKR. Although outpatient physiotherapy has been demonstrated to be effective in improving short-term functional outcomes after TKR [19, 20], it has not been implemented into routine clinical practice. A contributing factor to this is likely to be the lack of evidence about long-term clinical effectiveness and cost-effectiveness of this service. Our study will provide this evidence base for decision-making, inform the development of guidelines for post-discharge care to this growing patient group, and ultimately lead to improved patient outcomes after TKR, with an efficient use of health care resources. This research aims to provide evidence needed to guide decisions by clinicians, policy-makers and patients and inform commissioning of services to ensure all patients receive the best physiotherapy care after TKR.

## Trial status

This trial began patient recruitment in March 2015.

## Abbreviations

CEA, cost-effectiveness analysis; CEAC, cost-effectiveness acceptability curve; CUA, cost-utility analysis; GPs, general practitioners; HADS, Hospital Anxiety and Depression Scale; ICER, incremental cost-effectiveness ratio; INMB, incremental net monetary benefit; KOOS, Knee Injury and Osteoarthritis Outcome Score; LEFS, Lower Extremity Functional Scale; MCID, minimal clinically important difference; MI, multiple imputation; NHS, National Health Service; OA, osteoarthritis; PEP-R, Patient Experience Partnership in Research; PSS, Personal Social Services; QALYs, quality-adjusted life years; RCT, randomised controlled trial; SD, standard deviation; TKR, total knee replacement
